# Teaching Methods that Promote Medical Student Attendance: A Scoping Review

**DOI:** 10.1007/s40670-025-02535-0

**Published:** 2025-12-22

**Authors:** Jenny R. Mills, Pat W. Whitworth, Jamie G. Shaffer, Jeff L. Jackson, Sarah E. Keil

**Affiliations:** 1 Thomas F. Frist, Jr. College of Medicine, Belmont University, Nashville, TN USA; 2 Alice L Walton School of Medicine, AR Bentonville, USA

**Keywords:** Attendance, Undergraduate medical students, Active learning, Faculty development

## Abstract

**Background:**

Medical student class attendance, especially traditional lectures, is on the decline. As faculty explore a variety of pedagogical approaches, understanding how to encourage attendance and engagement is critical. The purpose of this scoping review is to explore the literature to better understand the teaching methods that promote attendance.

**Methods:**

This scoping review was conducted as advised in the Joanna Briggs Institute (JBI) Manual for Evidence Synthesis. Five databases were systematically searched for primary studies on teaching methods that motivated undergraduate medical students to attend or not attend in-person or online synchronous class sessions. A total of 1313 articles were identified from database and hand searching; then, 318 full records were screened, and 31 were included in the review.

**Results:**

A variety of teaching methods may motivate medical students’ class attendance, including both well-structured, engaging lectures and active learning activities. The perceived quality of lecturers was a determining factor in lecture attendance, but the teaching method that motivated more consistent attendance was active learning. The review found that the availability of recorded lectures does not reduce attendance rates and may lead to greater engagement and attendance.

**Conclusions:**

To address attendance declines, institutions should adopt a multifaceted strategy that includes faculty development in active learning and student flexibility through the provision of online course materials. Enhancing the perceived value of in-person lectures through engagement and interaction is crucial. Future research and policy should balance flexibility with meaningful engagement, ensuring attendance is motivated by educational value rather than obligation.

## Introduction

### Rationale

The landscape of medical education is continuously evolving, with educators exploring a variety of pedagogical approaches to enhance student learning and engagement. While traditional lectures have long been a cornerstone of medical curricula, student attendance at these sessions decreases as they progress through the first year and into the second year [1–5], often below levels desired by faculty. Data from the most recent AAMC Year 2 Questionnaire show that students tend to cluster in the extremes regarding attendance, with most attending in-person class either “almost never” (19.6%) or “most of the time” (39.9%), indicating widely differing philosophies across institutions about the value of class attendance [6]. Understanding how to encourage medical student attendance and engagement with in-person and/or synchronous online learning is critical. Despite the increasing diversity of teaching methods, a clear understanding of which approaches best promote student attendance remains elusive. A recent study of student perceptions and expectations around lecture attendance found that medical students’ decisions about which learning activities to attend are influenced by many factors, including preferences for certain teaching methods and presentation styles, as well as perceived relevance to board examinations and future careers [1].

In this scoping review, we aim to map the extent, range, and nature of the diverse primary literature on the range of teaching methods used to promote class attendance among undergraduate medical students, and to explore the relationship between student engagement and attendance. By providing clear definitions and identifying areas where further investigation is needed, as well as summarizing and disseminating key findings, this review can help advance our understanding of how teaching methods influence student participation in medical education. We hope the findings are helpful to faculty and administrators alike, as many in medical education are grappling with attendance policy questions.

This scoping review was conducted as advised in the Joanna Briggs Institute (JBI) Manual for Evidence Synthesis [7] and was reported according to the Preferred Reporting Items for Systematic Reviews extension for Scoping Reviews (PRISMA-ScR) checklist [8].

### Review Question

How do teaching methods influence class attendance or non-attendance among undergraduate medical students?

### Objectives

This scoping review aims to assess the extent of the literature available to address the following questions:

- What teaching methods have been used to promote in-person and/or synchronous online class attendance among undergraduate medical students?
- What are the effects of these teaching methods on class attendance among undergraduate medical students?

### Definitions

The transition in medical education from traditional didactic lectures to multi-modal and digital instruction began at the turn of the twenty-first century, driven by the emergence of learning management systems and multimedia tools that offered new possibilities for content delivery and learner engagement [9]. While new technologies introduced a wide array of teaching methods and modalities, from asynchronous video lectures and interactive modules to blended formats, lecture remains a foundational component of medical education, whether delivered in-person in a classroom or online through recordings or by live-stream. This proliferation of instructional approaches has led to conceptual ambiguity, however, with terms used interchangeably or inconsistently. Clear definitions are therefore essential to distinguish between the strategies educators use (methodologies) and the formats through which instruction is delivered (modalities). We will use the following defined terms throughout the article.

- **Teaching methods**—The techniques or strategies that teachers use to deliver content, facilitate learning, and meet the course objectives

The two primary teaching methods compared in this study that relate to attendance are lecture and active learning.

- **Lecture**—“An instruction or verbal discourse by a speaker before a large group of learners.” Lectures are instructor-centered and are synonymous with “didactic” [10].
- **Active learning**—Requires students to do something or engage in some activity that will prompt them to make their own meaning of the content being taught. Active learning can encompass a broad range of student-centered activities such as classroom assessment techniques, audience response systems, and case-based instruction [11, 12].

There are also different modalities and ways of delivering instruction.

- **In-person**—Where the instructor and students are in the same physical space for the learning session. Synonymous with “face-to-face” [13].
- **Online**—The use of digital platforms and technology to deliver instructional content and facilitate learning interactions remotely. Can include synchronous and asynchronous formats [13].
- **Blended**—Combining in-person and online modalities to enhance flexibility, engagement, and accessibility [13].
- **Flipped classroom learning**—A type of blended learning in which asynchronous materials are provided, typically digitally, prior to in-person application in the classroom [13].
- **Synchronous**—Occurs when learners are learning together at the same time. Can occur in both in-person and online formats [13].
- **Asynchronous**—Occurs when learners can participate on their own, from anywhere at any time. Can include pre-readings, audio or video recordings, discussion boards, or tutorials delivered via online modules, email, or Learning Management System [13].

### Eligibility Criteria

Studies were included if they focused on the following criteria:

- Primary research
- Undergraduate medical students
- Teaching methods as motivating or non-motivating factors leading to class attendance or non-attendance
- In-person or online synchronous instruction in a classroom or lab setting

Studies were excluded for the following reasons:

- More than 20 years old
- Studies where attendance is mentioned but it is not one of the main subjects
- Studies focused on attendance policies only rather than teaching methods
- Studies that focused solely on faculty perceptions of teaching methods and attendance without considering the impact/perceptions/attitudes of students
- Non-medical students
- Non-regular classroom settings (e.g., clinical setting)

## Methods

### Information Sources and Search Strategy

To comprehensively search the relevant literature, the following bibliographic databases were searched: MEDLINE via PubMed, Embase, Education Source via EBSCO, ERIC via Ovid, and Scopus. The original searches were conducted on May 3, 2023, and were filtered by publication date, from 2003 to 2023. A limit to articles published in the last 20 years was selected as the time frame when attendance patterns began to change in medical education as a result of emerging technologies such as the web, learning management systems, blogs, social media, and audio and video recording technology [14–16]. The search was updated on June 24, 2024, to capture any new articles published from 2023 through June 24, 2024. The keywords were discussed and refined by the research team, and the search strategy was developed and run by a librarian on the team. Only studies published in English were included, as that is the only language spoken by all members of the research team. The final search strategy for MEDLINE via PubMed can be found in the Appendix.

In addition to the database searches, hand searching of relevant medical education journals was completed. The journals were identified as ones read most often by members of the research team for studies on medical education and included *Academic Medicine*, *BMC Medical Education*, *Medical Education*, *Teaching and Learning in Medicine*, and *Medical Teacher*. The table of contents of each issue of each journal was hand searched for the same 20-year date range, January 2003 through June 2024.

### Study Selection

Following the search, all identified citations were collated and uploaded into EndNote 21 (https://endnote.com/downloads/?srsltid=AfmBOorMJLKxgAMe3g7waaaLpnu1m01hxkccFwmn3EWppfAIygIeVmXQ) and duplicates were removed using the Bramer Method [17]. The results were then exported to Rayyan (https://www.rayyan.ai/), a web-based tool, for two rounds of screening. First, following a test of sample articles, titles and abstracts were screened independently by two reviewers from the research team for assessment against the inclusion criteria for the review. Second, two independent reviewers also screened the full text of each article. For all screenings, the reviewers met to discuss and resolve any discrepancies after each round.

### Data Charting

A preliminary data extraction form was developed following JBI’s recommendations on data extraction [7]. The form was used on a sample of articles and subsequently revised according to the identified needs of this review. Two reviewers independently examined each article and met to discuss any discrepancies. The final tool extracted data on the author, title of the article, date of publication, title of the journal, purpose of the study, method (quantitative or qualitative), specific method (focus group, questionnaire, etc.), population, modality, instructional method, discipline, if present, attendance policy, whether grades were attached to attendance, and findings. One author reviewed all extracted data to identify themes, and the group met to review and discuss.

### Data Synthesis

The reviewers quantified study characteristics including populations, year of medical school, study method (quantitative or qualitative), and specific method (focus group, questionnaire, etc.). Also, from the data, the primary teaching methods were identified, and specific aspects of those methods were further analyzed. Although not the main focus of the study, attendance policies were noted, if included in the studies, as were any measures of academic performance being influenced by attendance. Finally, studies related to the COVID-19 pandemic were identified, and the impact of the pandemic was analyzed in relation to patterns of attendance.

## Results

**Fig. 1:**
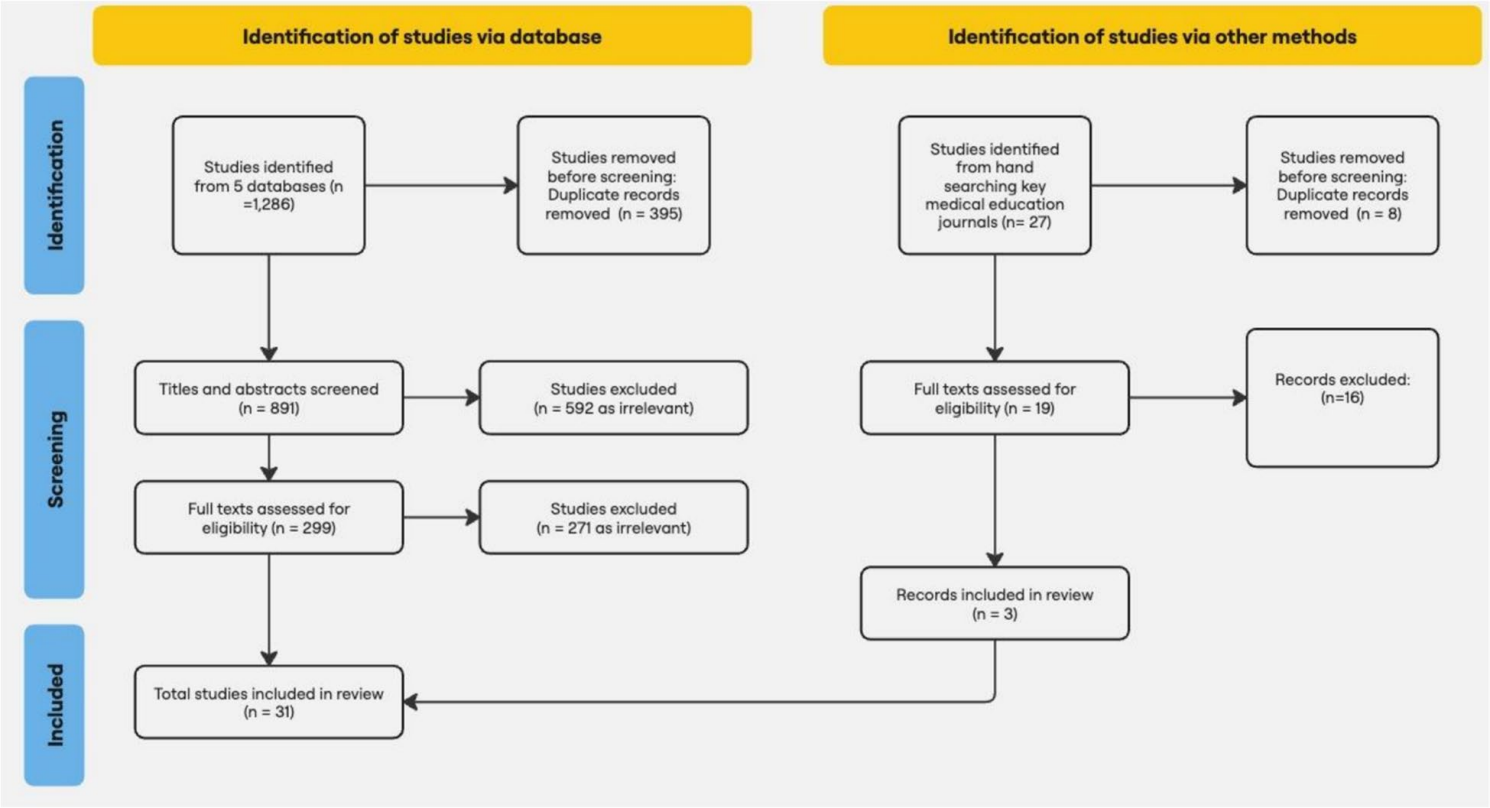
Flow diagram for the literature search and selection of articles for a scoping review of teaching methods that promote student attendance

A total of 1286 studies were identified from database searching and 27 from hand searching. Four hundred and three duplicates were removed, and 910 titles and abstracts were screened. Five hundred and ninety-two studies were removed as irrelevant or not meeting the inclusion criteria, and full texts were assessed for 318 studies. Thirty-one studies met the inclusion criteria and were included in the review. See Fig. 1 for a flow diagram of this process.

**Fig. 2:**
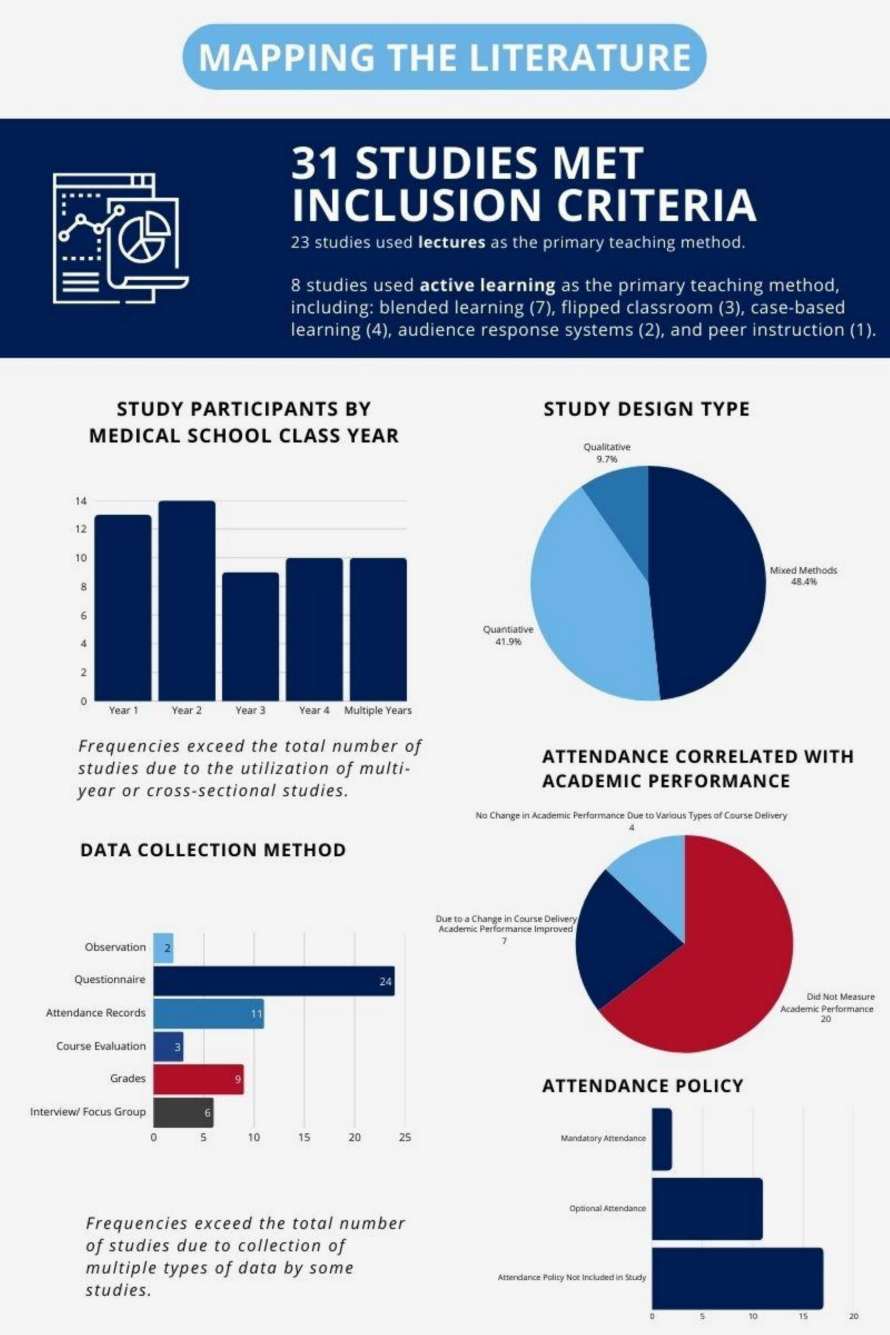
Mapping the literature

After compiling the 31 studies meeting the inclusion criteria, these studies were further considered in terms of study participants, study design type, data collection method, and teaching method (see Fig. 2 for a visual of how the literature was mapped).

The institutions that conducted studies on attendance did so across a range of student years. The most common were the first (13) and second (14) years, with seven of the studies using both first- and second-year students as their population. There were 10 overall studies that utilized multiple student years in their study design.

Mixed methods (15) was the most common method, followed by quantitative (13) and qualitative (three). Twenty-four of the studies collected data with questionnaires designed for their particular studies, while others used existing data sources: attendance records (11), grades (nine), and course evaluations (three).

Studies were included regardless of whether attendance was mandatory or voluntary, and for 17 studies, this was unclear because there was no mention of an attendance policy. Attendance was voluntary in 11 studies, and in two studies, attendance was mandatory.

The focus of this review was not on the effects of attendance on academic performance, but 11 studies included some measure of academic performance as a result of a change in teaching method(s) and/or increased attendance. Twenty studies made no mention of academic performance.

The manuscripts revealed two primary teaching methods—lecture and active learning. Twenty-three articles primarily focused on lectures or didactic teaching [1–5, 18–36]. Eight articles utilized active learning in their studies [16, 36–42], with the majority incorporating blended learning as the foundation for their approach [16, 36–40, 42]. Approaches used within the studies included flipped classroom [36, 37, 42] and case-based learning [16, 38–40]. Additionally, audience response systems [36, 41] and peer instruction [37] were prominently included as teaching methods.

### Lecture

It is common for students to start medical school attending in-person lectures; however, as they progress through the first year and into the second year, their attendance declines [1–5]. This is for both in-person and online lecture sessions. Lectures may be delivered in-person or online, but thematically, they have similar outcomes. Students choose not to attend based on perceived usefulness related to their time and the quality of the session. Studies that identified personal characteristics discovered that female students attend more live lectures [18] along with students applying to less competitive specialties [1]. The perceived quality of the lecturer, including their preparation and presentation skills, has the most significant influence on students attending in-person lectures. This includes their ability to explain and communicate complex concepts and their preparation of the content [1, 18–24]. This is further confirmed when targeted course improvements yielded higher satisfaction and attendance [25]. In studies that utilized in-class activities in conjunction with lectures, attendance was improved [1, 26, 27]. For example, student-centered lectures with clicker-based formative assessments were viewed more positively than passive, instructor-centered lectures [1, 37]. In one study, attendance was further incentivized by the possibility of earning “clicker points” in class [2]. In studies where students had the option between online and in-person lectures, the option for online resources such as audio or video recorded lectures or textual course materials made available online such as lecture notes did not negatively impact student attendance [1, 5, 28–32]. Interestingly, in one study, students had flexibility in their choice of lecture format: synchronous in-person, online, or asynchronous recordings, and 58% preferred synchronous in-person lectures because they wanted direct interaction with peers and instructors [2]. Furthermore, seven studies found that in blended modalities, if the online material is organized well, it can increase engagement in the synchronous sessions [1, 5, 29–33]. Two studies found that ease of access improved attendance in online courses [34, 35].

### Active Learning

Active learning has a positive impact on improving attendance and the learning environment regarding in-person learning [16, 36–42]. It provides increased opportunities to engage with classmates [16, 36, 39], the course content [41], and instructors [42]. Flipped-classroom learning led to increased attendance and engagement. In one study, providing notes and images related to the course content in advance allowed instructors to spend less time on conveying basic concepts and made the in-person sessions more active and participatory [30].

### Academic Performance

While the correlation between academic performance and attendance was not the focus of this review, 11 studies included some measure of academic performance in addition to student motivation for attendance [2, 3, 5, 22, 26, 27, 35, 37–40]. Five studies found no change in academic performance due to either a change in teaching method or in course modality (online vs. in-person) [2, 3, 5, 39, 40]. Three studies compared in-person and online delivery of lectures and found no correlation between modality, attendance rates, and grades [2, 3, 5]. One study found that the introduction of case-based learning led to increased attendance but not to test scores when compared with lectures [39], and one study found no difference in exam results between problem-based learning delivered in-person compared to a blended format [40]. Six studies did find a correlation between a change in teaching method or modality and academic performance. In one study comparing the same course delivered in-person and then remotely during the pandemic, students’ final scores were higher for the online learning group. Those students also attended the online synchronous lectures more regularly than the in-person group [35]. In another study, students with access to online course materials via Moodle had higher attendance rates to the in-person lectures and higher formative and summative grades [26]. Four studies found that incorporating active learning strategies led to improved academic performance. Flipped classroom learning, team-based learning, and small group learning all increased performance when compared with traditional and passive in-person lectures [22, 27, 37, 38].

### COVID-19 Interventions

Six articles within the pooled data indicated their intervention was a response to the COVID-19 pandemic [2, 22, 24, 33–35]; however, inconsistencies regarding factors such as teaching method, modalities, attendance policies, and student performance and engagement outcomes limited attribution of findings specifically to pandemic-related efforts.

## Discussion

This scoping review examined how different teaching methods influence medical student attendance, identifying key patterns related to lecture-based learning, active learning, and external factors such as the COVID-19 pandemic. Overall, studies indicated that student attendance generally declines throughout the first and second years in medical school, particularly in lecture-based formats, regardless of whether sessions are delivered in-person or online. The decline is largely driven by perceived usefulness, with students opting out of sessions they do not find valuable in terms of content delivery, engagement, or instructional quality.

### Lectures

Lecture-based learning remains the predominant mode of instruction, with most studies focusing on its effectiveness. Findings suggested that well-structured, engaging lectures delivered by skilled and prepared instructors can positively impact attendance. The perceived quality of lecturers, including their ability to explain complex concepts and engage students, emerged as a crucial determinant of attendance. Interestingly, most studies found that the availability of asynchronous course materials such as audio or video recorded lectures, slides, or notes did not negatively impact student attendance and, if well-organized and easy for students to access, may actually increase attendance to synchronous lectures, whether delivered in person or online. Over 40% of the students surveyed in the most recent Y2Q survey indicated that they use online recorded lectures “most of the time,” suggesting that they heavily value such virtual content in learning [6]. To counteract attendance declines, institutions should prioritize faculty development programs to improve lecture quality, ensuring that instructors are motivated and equipped to deliver engaging and effective sessions. Providing training in student-centered teaching methods and interactive instructional design can help make in-person attendance more valuable for students.

### Active Learning

In contrast, active learning methodologies, including various degrees of flipped classrooms, case-based learning, and blended learning, showed a more consistent positive effect on attendance. These approaches increase student engagement with peers and instructors, making the in-person experience more valuable. The number of students who attend in-person class “most of the time” and those who utilize online content “most of the time” are similar (37% v. 41%, respectively), suggesting that blended learning has become a common practice [6]. Even within didactic instruction, integrating interactive pre-class resources appears to enhance attendance and engagement levels. In one study, video lectures incorporating embedded quizzes functioned as flipped classroom instruction, requiring quiz completion to advance and enabling faculty to monitor the time students spent engaging with the material. This instructional approach was associated with sustained in-person attendance throughout the module, contrasting with previously observed declines, except in components not utilizing the flipped format [37]. Given the positive impact of active learning methodologies on attendance, expanding the use of these techniques could foster greater student engagement. Additionally, leveraging student feedback to tailor instructional approaches may further enhance attendance. Regular assessment of student preferences, perceived barriers to attendance, and perceptions of instructional effectiveness can help educators refine their teaching strategies and create a more engaging learning environment.

### Academic Performance

Studies that included some measure of academic performance related to attendance and a change in teaching methods or modality produced mixed results, with some reporting improvements and some reporting no change in educational outcomes. This is consistent with other findings in the literature. For example, a systematic review found that incorporating innovative teaching methods and blended learning improved attendance, but when exploring the relationship between attendance and academic performance, the results were mixed. The majority of the studies in the review noted a positive correlation, but noted that many factors could influence these outcomes [43].

#### COVID-19

While this review did consider methodologies that were specifically implemented due to the COVID-19 pandemic, there were mixed results among these studies and no consistent themes emerged regarding impact on attendance, limiting the ability to draw broad conclusions.

### A Multifaceted Strategy

Although no single approach guarantees high attendance, a multifaceted strategy that includes faculty development to improve lecture quality, active learning, and structured online resources may help mitigate attendance declines. Institutions should focus on enhancing the perceived value of in-person sessions through engaging instruction and interactive methodologies. Future research and policy adjustments should aim to balance flexibility with meaningful engagement, ensuring that attendance is driven by educational value rather than obligation.

### Strengths and Limitations

This scoping review identified a substantial body of literature examining the relationship between teaching methods and student attendance in undergraduate medical education. As such, there are various strengths and limitations that must be acknowledged. The review included 31 studies spanning a 21-year period (2003–2024), reflecting the growing interest in understanding and improving attendance in medical education. The range of research covered various teaching methods, including lecture-based and active learning approaches, as well as blended learning models that combine in-person and online components. However, while the studies examined broad categories, variations within each approach (e.g., different implementations of the same “type” of active learning) may influence attendance differently. Moreover, while some studies explored the impact of blended and online learning environments, the specific implementation of such programs can vary greatly and may affect student attendance in a myriad of ways. This finding indicates the need for more targeted research in this area.

Study populations were fairly balanced, with representation across different years of undergraduate medical training. First- and second-year students were the most frequently studied populations, reflecting the common observation that attendance tends to decline as students progress through their medical education. The focus on undergraduate medical students allows for deeper insights into this particular population, but excluding perspectives from faculty members, administrators, or postgraduate learners may limit understanding of institutional policies, faculty expectations, and broader systemic factors influencing attendance that could provide a more comprehensive picture.

There was a good amount of methodological diversity throughout the reviewed literature. Studies employed a mix of qualitative, quantitative, and mixed methods approaches, with the most common methods being questionnaires (24 studies), attendance records (11 studies), and grade records (nine studies). This variety is both a strength and limitation of this study. While it allows for a broader exploration of student motivations and attendance patterns, it also makes it challenging to synthesize findings and identify consistent patterns. Given the predominance of cross-sectional data in the articles available, longitudinal studies would be invaluable in tracking the evolution of attendance patterns throughout medical school and their correlation with academic performance, career choices, and other long-term outcomes. Furthermore, additional qualitative research could offer rich insights into student and faculty perceptions of attendance, the motivations behind attendance decisions, and the perceived value of various instructional approaches.

In terms of quality, the reviewed studies demonstrate a range of research rigor. The reliance on self-reported attendance data in many studies introduces potential bias, as students may overestimate or underreport their attendance behaviors. Such study designs limit the ability to draw causal inferences about the relationship between teaching methods and attendance. As noted, few studies accounted for institutional or contextual factors, which may influence generalizability.

### Future Directions

The findings from this scoping review identified a number of gaps in the literature on this topic, leading to several avenues for future research. One key area is further investigation into the role of faculty development in shaping attendance behaviors. Given that instructor quality and engagement significantly influence attendance, future studies should examine how faculty training in pedagogy, student engagement, and technology integration affects student perceptions of lecture quality.

Additionally, the relationship between attendance and academic performance warrants further study. While attendance is often assumed to correlate with learning outcomes, more robust research is needed to determine the extent to which higher rates of attendance, as well as increased student engagement/participation, affect various academic outcome measures.

Another promising area for research is the impact of blended learning environments that combine online and in-person components, taking the strengths of both approaches to enhance flexibility, engagement, accessibility, and student satisfaction. There is evidence that blended learning leads to better academic outcomes than either in-person or online alone [13]. Understanding how students navigate these options, and what factors encourage them to attend in-person sessions when online alternatives exist, could inform instructional design and curriculum decisions.

Finally, institutional policies around attendance should be further explored. While this review focused on teaching methods, broader structural factors such as attendance policies, grading incentives, and institutional culture likely play a role in shaping attendance behaviors. Future research should examine the degree to which different policy approaches influence attendance and meaningful engagement.

## Conclusion

This scoping review summarizes and explores the extent of research that examines the role of teaching methods in medical student attendance, with active learning strategies generally promoting higher levels of engagement compared to traditional lectures. However, the quality of instruction, perceived relevance of content, and availability of online resources all influence attendance decisions. While the shift toward active learning appears promising, further research is needed to explore the effects of a wider variety of specific techniques. Studies examining the impact of faculty development initiatives and the strategic use of online resources may be needed in order to guide institutions in creating a more engaging and effective learning environment that encourages attendance while maintaining flexibility. By addressing these gaps, future researchers can assist medical educators in developing more effective strategies to encourage meaningful student participation in both in-person and online learning environments.

## Data Availability

The data extraction form results are available from the authors by request.

## Appendix

### Search Strategy

Search Strategy for MEDLINE via PubMed on May 3, 2023. Filtered by publication date, from 2003 to 2023, and re-run on June 24, 2024, filtered from 2023 to June 24, 2024.

(((Attendance[tiab] OR "Absenteeism"[Mesh]) AND (class[tiab] OR classroom[tiab] OR classes[tiab] OR policy[tiab] OR policies[tiab] OR mandatory[tiab] OR lecture[tiab] OR required[tiab])) AND ("Medical students"[tiab] OR "undergraduate medical education"[tiab] OR "medical education"[tiab] OR "medical school"[tiab] OR "Students, Medical"[Mesh] OR "Education, Medical, Undergraduate"[Mesh] OR "Schools, Medical"[Mesh] OR "Education, Medical"[Mesh])) AND ("Academic performance"[tiab] OR "academic achievement"[tiab] OR "academic success"[tiab] OR "academic outcomes"[tiab] OR "academic assessment"[tiab] OR grades[tiab] OR “Academic Success"[Mesh] OR "Academic Performance"[Mesh] OR "Academic Failure"[Mesh] OR "Educational Measurement"[MeSH] OR learning[tiab] OR USMLE[tiab] OR choice[tiab] OR “exam success”[tiab] OR perceptions[tiab] OR satisfaction[tiab] OR expectations[tiab] OR perspectives[tiab] OR engagement[tiab] OR motivation[tiab] OR participation[tiab] OR well being[tiab])).

## References

[1] Emahiser J, Nguyen J, Vanier C, Sadik A. Study of live lecture attendance, student perceptions and expectations. Med Sci Educ. 2021. 10.1007/s40670-021-01236-8. 10.1007/s40670-021-01236-8 PMC8368907 34457920

[2] Faner MA, Ritchie RP, Ruger KM, Waarala KL, Wilkins CA. Student performance in medical biochemistry and genetics: comparing campus-based versus zoom-based lecture delivery. BMC Med Educ. 2022. 10.1186/s12909-022-03873-y. 10.1186/s12909-022-03873-y PMC9668392 36384548

[3] Ikonne U, Campbell AM, Whelihan KE, Bay RC, Lewis JH. Exodus from the classroom: student perceptions, lecture capture technology, and the inception of on-demand preclinical medical education. J Am Osteopath Assoc. 2018. 10.7556/jaoa.2018.174. 10.7556/jaoa.2018.174 30476993

[4] Mattick K, Crocker G, Bligh J. Medical student attendance at non-compulsory lectures. Adv Health Sci Educ Theory Pract. 2007. 10.1007/s10459-005-5492-1. 10.1007/s10459-005-5492-1 17041787

[5] Schöbel T, Zajonz D, Melcher P, Lange J, Fischer B, Heyde C-E, et al. Podcasts as a teaching tool in orthopaedic surgery: is it beneficial or more an exemption card from attending lectures? Orthopade. 2021. 10.1007/s00132-020-03956-y. 10.1007/s00132-020-03956-y PMC8189972 32749511

[6] AAMC. Year two questionnaire (Y2Q). https://www.aamc.org/data-reports/students-residents/report/year-two-questionnaire-y2q. Accessed 29 Aug 2025.

[7] Aromataris E, Munn Z. JBI manual for evidence synthesis. JBI. 2020. https://synthesismanual.jbi.global. Accessed 16 April 2025.

[8] Tricco AC, Lillie E, Zarin W, O’Brien KK, Colquhoun H, Levac D, et al. PRISMA extension for scoping reviews (PRISMA-ScR): checklist and explanation. Ann of Intern Med. 2018. 10.7326/M18-0850. 10.7326/M18-0850 30178033

[9] Chowdhury PN, Vaish A, Puri B, Vaishya R. Medical education technology: past, present and future. Apollo Medicine. 2024. 10.1177/09760016241256202.

[10] MedBiquitous Curriculum Inventory Working Group Standardized Vocabulary Subcommittee. Curriculum Inventory standardized instructional and assessment methods and resource types. MedBiquitous: Association of American Medical Colleges. 2016. https://www.medbiq.org/media/3241/download Accessed 18 Aug 2025.

[11] Bucklin B, Asdigian N, Hawkins J, Klein U. Making it stick: use of active learning strategies in continuing medical education. BMC Med Educ. 2021. 10.1186/s12909-020-02447-0. 10.1186/s12909-020-02447-0 PMC7798232 33430843

[12] Graffam B. Active learning in medical education: strategies for beginning implementation. Med Teach. 2007. 10.1080/01421590601176398. 10.1080/01421590601176398 17538832

[13] MacNeill H, Masters K, Nemethy K, Correia R. Online learning in health professions education. Part 1: teaching and learning in online environments: AMEE guide no. 161. Med Teach. 2024. 10.1080/0142159X.2023.2197135. 10.1080/0142159X.2023.2197135 37094079

[14] Weller M. The rise and development of digital education. In: Zawacki-Richter O, Jung I, editors. Handbook of open, distance and digital education. Singapore: Springer; 2022. 10.1007/978-981-19-0351-9_5-1

[15] Freeburg T, Chang TP. Digital technology for medical education. In: Adirim T, editor. Digital health, AI and generative AI in healthcare. Cham: Springer; 2025. 10.1007/978-3-031-83526-1_16

[16] Marchevsky AM, Relan A, Baillie S. Self-instructional, “virtual pathology” laboratories using web-based technology enhance medical school teaching of pathology. Hum Pathol. 2003. 10.1016/S0046-8177(03)00089-3. 10.1016/s0046-8177(03)00089-3 12792914

[17] Bramer WM, Giustini D, de Jonge GB, Holland L, Bekhuis T. De-duplication of database search results for systematic reviews in EndNote. J Med Libr Assoc. 2016. 10.3163/1536-5050.104.3.014. 10.3163/1536-5050.104.3.014 PMC4915647 27366130

[18] Gupta A, Saks NS. Exploring medical student decisions regarding attending live lectures and using recorded lectures. Med Teach. 2013. 10.3109/0142159X.2013.801940. 10.3109/0142159X.2013.801940 23869431

[19] Akturan S, Sevım M, Erzık C, Yegen B, Gulpınar MA. Rethinking large group lectures – how far in this format? Marmara Med J. 2022. 10.5472/marumj.1121353.

[20] Aqahtani T, Albalawi A, Alotaibi J, Alshareef A, Alrasheed T, Mirghani H, et al. Chronotype, daytime sleepiness, and related factors effects on skipping classroom among medical students in Tabuk. Sudan J Med Sci. 2023. 10.18502/sjms.v18i2.13601.

[21] Billings-Gagliardi S, Mazor KM. Student decisions about lecture attendance: do electronic course materials matter? Acad Med. 2007. 10.1097/ACM.0b013e31813e651e. 10.1097/ACM.0b013e31813e651e 17895696

[22] Liu Z-P, Liu S-H, Dai X, Chen J, Guo Q-F, Zhang D-X. Block-based teaching method based on cybernetics: a trial with 115 Chinese undergraduate medical students. BMC Med Educ. 2023. 10.1186/s12909-023-04643-0. 10.1186/s12909-023-04643-0 PMC10494328 37691113

[23] Qureshi MI, Ahmad A. Medical students’ perspective on absenteeism and its remedies. Pak Armed Forces Med J. 2019;69(2):Article 2.

[24] Schick G, McWhorter D. Instructor methods and curricular effects on students’ value of lectures. Med Sci Educ. 2022. 10.1007/s40670-021-01459-9. 10.1007/s40670-021-01459-9 PMC8638641 34877072

[25] Kouz K, Eisenbarth S, Bergholz A, Mohr S. Presentation and evaluation of the teaching concept “ENHANCE” for basic sciences in medical education. PLoS ONE. 2020. 10.1371/journal.pone.0239928. 10.1371/journal.pone.0239928 PMC7523967 32991616

[26] Popovic N, Popovic T, Rovcanin Dragovic I, Cmiljanic O. A moodle-based blended learning solution for physiology education in Montenegro: a case study. Adv Physiol Educ. 2018. 10.1152/advan.00155.2017. 10.1152/advan.00155.2017 29357268

[27] Soyebi K. Changing students’ performance in and perception of radiology. Med Educ. 2008. 10.1111/j.1365-2923.2008.03076.x. 10.1111/j.1365-2923.2008.03076.x 18412897

[28] Cardall S, Krupat E, Ulrich M. Live lecture versus video-recorded lecture: are students voting with their feet? Acad Med. 2008. 10.1097/ACM.0b013e31818c6902. 10.1097/ACM.0b013e31818c6902 19202495

[29] Franklin DS, Gibson JW, Samuel JC, Teeter WA, Clarkson CW. Use of lecture recordings in medical education. Med Sci Educ. 2011. 10.1007/BF03341590.

[30] Gómez-Arbonés X, Ferreira A, Piqué M, Roca J, Tomás J, Frutos JL, et al. Short communications, a cardiological web as an adjunct to medical teaching: prospective analysis. Med Teach. 2004. 10.1080/01421590310001653991. 10.1080/01421590310001653991 15203530

[31] Topale L. The strategic use of lecture recordings to facilitate an active and self-directed learning approach. BMC Med Educ. 2016. 10.1186/s12909-016-0723-0. 10.1186/s12909-016-0723-0 PMC4983083 27520704

[32] Wang R, Mattick K, Dunne E. Medical students’ perceptions of video-linked lectures and video-streaming. Res Learn Technol 2010; https://journal.alt.ac.uk/index.php/rlt/article/view/872

[33] Bawazeer MA, Aamir S, Othman F, Alkahtani R. Students engagement using polls in virtual sessions of physiology, pathology, and pharmacology at King Saud bin Abdulaziz University for Health Sciences during COVID-19 pandemic: a cross-sectional study. BMC Med Educ. 2023. 10.1186/s12909-023-04253-w. 10.1186/s12909-023-04253-w PMC10121230 37085845

[34] Altaf R, Kling M, Hough A, Baig J, Ball A, Goldstein J, et al. The association between distance learning, stress level, and perceived quality of education in medical students after transitioning to a fully online platform. Cureus. 2022. 10.7759/cureus.24071. 10.7759/cureus.24071 PMC9097940 35573543

[35] Haydar A, Santos IS, Arcon LC, Martins MdeA, Tempski PZ, Zatz R. Remote vs. face-to-face activities in the teaching of renal pathophysiology in the context of social isolation during the early phase of the COVID-19 pandemic. Adv Physiol Educ. 2023. 10.1152/advan.00257.2022. 10.1152/advan.00257.2022 37615046

[36] Drumm B. Designing and reflecting on active learning and flipped classrooms for renal physiology. Int J Scholarsh Teach Learn. 2023. 10.20429/ijsotl.2023.17122.

[37] Hernández-Guerra M, Quintero E, Morales-Arráez DE, Carrillo-Pallarés A, Nicolás-Pérez D, Carrillo-Palau M, et al. Comparison of flipped learning and traditional lecture method for teaching digestive system diseases in undergraduate medicine: a prospective non-randomized controlled trial. Med Teach. 2021. 10.1080/0142159X.2020.1867312. 10.1080/0142159X.2020.1867312 33502276

[38] Inuwa IM, Al-Rawahy M, Roychoudhry S, Taranikanti V. Implementing a modified team-based learning strategy in the first phase of an outcome-based curriculum—challenges and prospects. Med Teach. 2012. 10.3109/0142159X.2012.668633. 10.3109/0142159X.2012.668633 22746967

[39] Kaur G, Rehncy J, Kahal KS, Singh J, Sharma V, Matreja PS, et al. Case-based learning as an effective tool in teaching pharmacology to undergraduate medical students in a large group setting. J Med Educ Curric Dev. 2020. 10.1177/2382120520920640. 10.1177/2382120520920640 PMC7223199 32435693

[40] Servos U, Reiß B, Stosch C, Karay Y, Matthes J. A simple approach of applying blended learning to problem-based learning is feasible, accepted and does not affect evaluation and exam results—a just pre-pandemic randomised controlled mixed-method study. Naunyn-Schmiedebergs Arch Pharmacol. 2023. 10.1007/s00210-022-02306-3. 10.1007/s00210-022-02306-3 PMC9581769 36264299

[41] Tuma F, Majeed H, Blebea J, Nassar A, Durchholz WC, Schofield S. The educational value of an audience response system use in an Iraqi medical school. BMC Med Educ. 2022. 10.1186/s12909-022-03381-z. 10.1186/s12909-022-03381-z PMC9040241 35473705

[42] Vavasseur A, Muscari F, Meyrignac O, Nodot M, Dedouit F, Revel-Mouroz P, et al. Blended learning of radiology improves medical students’ performance, satisfaction, and engagement. Insights Imaging. 2020. 10.1186/s13244-020-00865-8. 10.1186/s13244-020-00865-8 PMC7188751 32347421

[43] Nagappan PG, Tan SRX, Absar S, Brown S, Sayers S, McManus A, et al. Changes in medical student attendance at in-person teaching sessions: a systematic review. BMJ Open. 2025. 10.1136/bmjopen-2024-091768. 10.1136/bmjopen-2024-091768 PMC12090850 40389321

